# Supplementary material for: Non-Invasive Nanometer Resolution Assessment of Cell–Soft Hydrogel System Mechanical Properties by Scanning Ion Conductance Microscopy
Source: Int J Mol Sci. 2024 Dec 16;25(24):13479. doi: 10.3390/ijms252413479 (PMC11678895; doi:10.3390/ijms252413479)
Supplement: Supplementary file 1 [file ijms-25-13479-s001.zip › ijms-3369570-supplementary.pdf]

Supplementary Materials

Non-Invasive Nanometer Resolution Assessment of Cell–Soft Hydrogel System Mechanical Properties by Scanning Ion Conductance Microscopy

Tatiana N. Tikhonova [1,*], Anastasia V. Barkovaya [1], Yuri M. Efremov [2], Vugara V. Mamed-Nabizade [3], Vasilii S. Kolmogorov [3,4], Peter S. Timashev [2,4,5], Nikolay N. Sysoev [1], Victor V. Fadeev [1], Petr V. Gorelkin [3], Lihi Adler-Abramovich [6,7,8], Alexander S. Erofeev [3] and Evgeny A. Shirshin [1,5,*]

[1] Department of Physics, M.V. Lomonosov Moscow State University, 1/2 Leninskie Gory, 119991 Moscow, Russia; anastasia.bark18@gmail.com (A.V.B.); nn.sysoev@physics.msu.ru (N.N.S.); victor_fadeev@mail.ru (V.V.F.)

[2] Institute for Regenerative Medicine, Sechenov University, 8-2 Trubetskaya St., 119991 Moscow, Russia; efremov_yu_m@staff.sechenov.ru (Y.M.E.); timashev_p_s@staff.sechenov.ru (P.S.T.)

[3] Laboratory of Biophysics, National University of Science and Technology MISIS, 4 Leninskiy prospekt, 119049 Moscow, Russia; vugara2003@yandex.ru (V.V.M.-N.); vskolmogorov@gmail.com (V.S.K.); peter.gorelkin@gmail.com (P.V.G.); erofeev.as@misis.ru (A.S.E.)

[4] Department of Chemistry, M.V. Lomonosov Moscow State University, 1/2 Leninskie Gory, 119991 Moscow, Russia

[5] World-Class Research Center "Digital Biodesign and Personalized Healthcare", Sechenov First Moscow State Medical University, 8-2 Trubetskaya St., 119991 Moscow, Russia

[6] Department of Oral Biology, The Goldschleger School of Dental Medicine, Faculty of Medical & Health Sciences, Tel Aviv University, Tel Aviv 6997801, Israel; lihiab@gmail.com

[7] The Center for Nanoscience and Nanotechnology, Tel Aviv University, Tel Aviv 6997801, Israel

[8] The Center for the Physics and Chemistry of Living Systems, Tel Aviv University, Tel Aviv 6997801, Israel

* Correspondence: tikhonova@physics.msu.ru (T.N.T.); eshirshin@gmail.com (E.A.S.); Tel.: +7-(495)-939-12-25 (T.N.T. & E.A.S.)

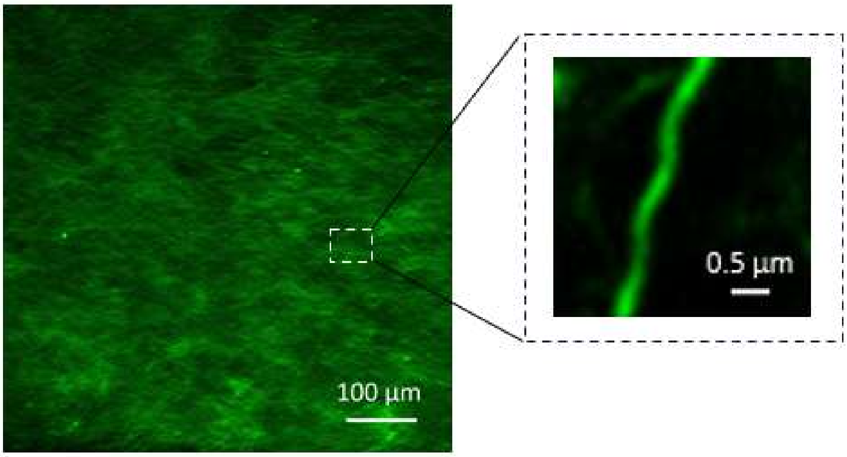

Figure S1. Fluorescence confocal microscopy image of self-assembly Fmoc-FF hydrogel structure obtained at 405 nm excitation following staining with 10 µM Thioflavin T.

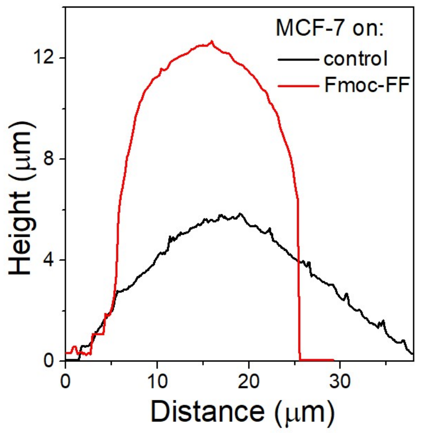

Figure S2. Profiles of MCF-7 cells placed on Petri dish (control) and Fmoc-FF hydrogel.

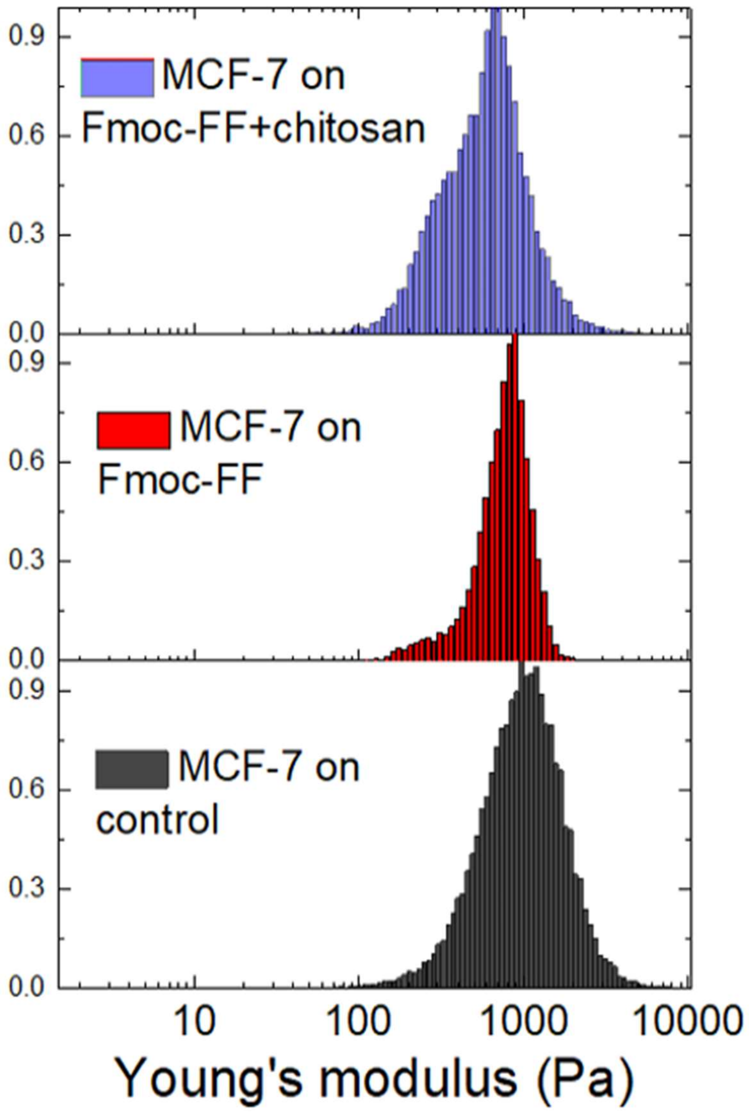

Figure S3. The histogram of Young's modulus distribution for MCF-7 cells placed on cultural Petri dish (control, black lines), Fmoc-FF hydrogel (red lines) and Fmoc-FF+chitosan hydrogel (violet lines).

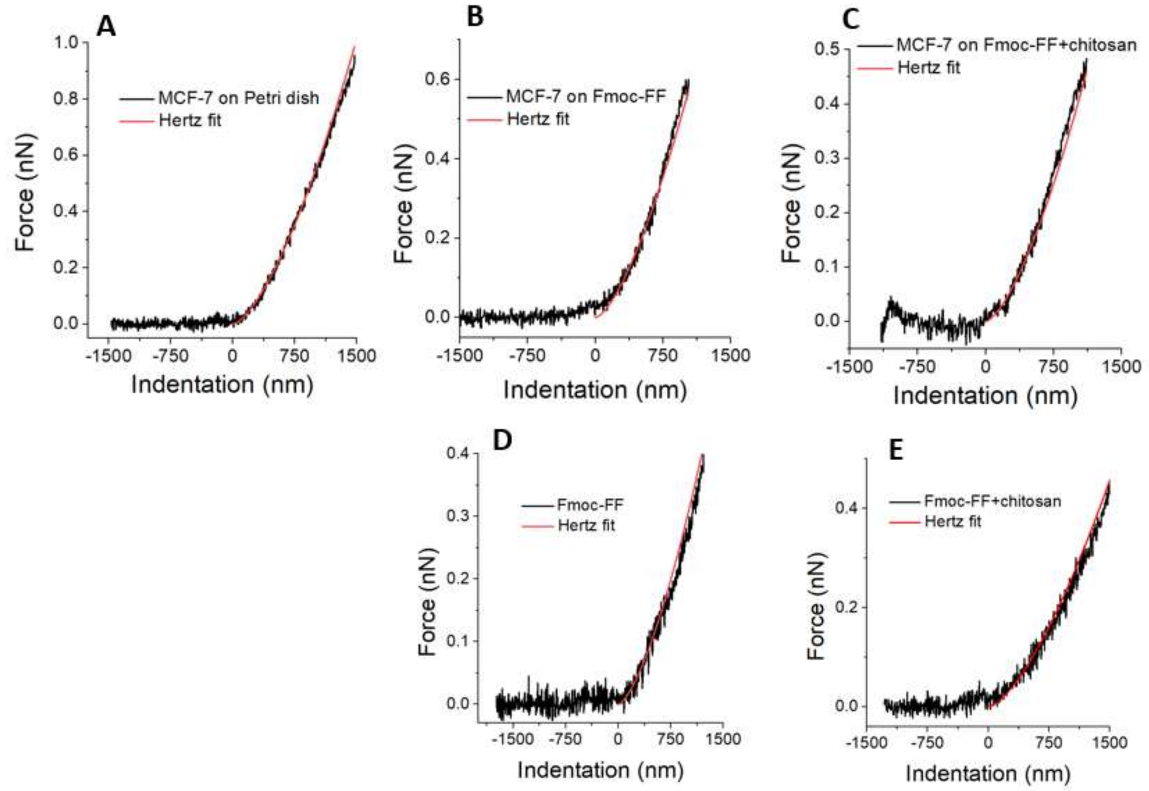

Figure S4. Force curves for (A) MCF-7 on Petri dish, (B) MCF-7 on Fmoc-FF hydrogel, (C) MCF-7 on Fmoc FF+chitosan hydrogel, (D) Fmoc-FF hydrogel, (E) Fmoc-FF+chitosan hydrogel.

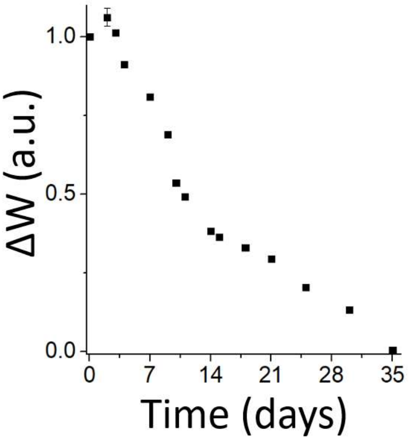

Figure S5. The degradation of Fmoc-FF hydrogel.

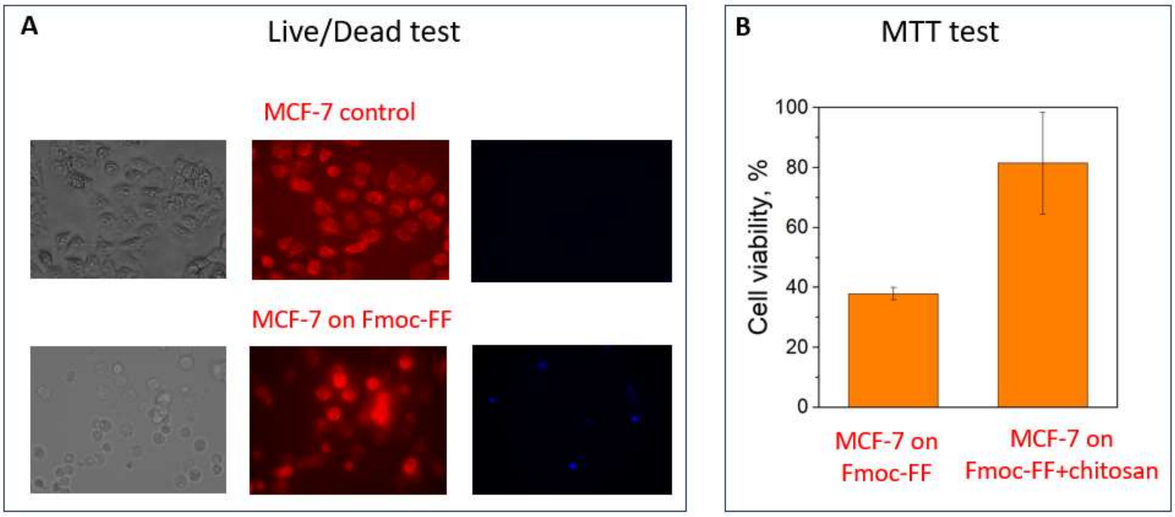

Figure S6. (A) Live-dead staining of MCF-7 cells after placing them on the Fmoc-FF hydrogels for 48 h. Red staining indicates live cells, blue staining indicates dead cells. (B) MTT test of MCF-7 cells on Fmoc-FF based hydrogels.

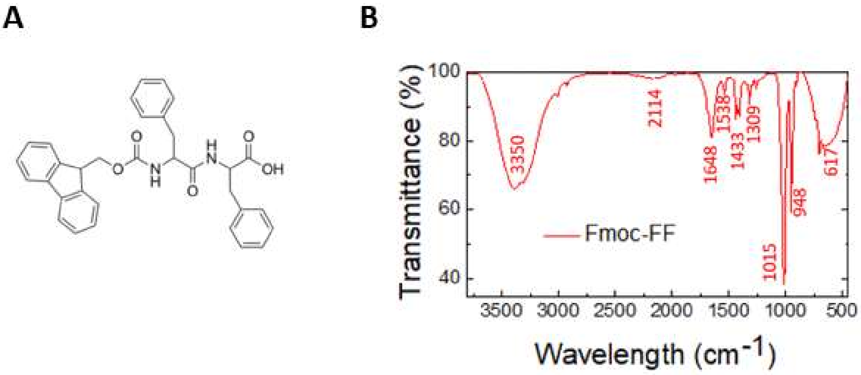

Figure S7. (A) Chemical structures of Fmoc-FF. (B) The FTIR analysis for Fmoc-FF hydrogel.

The FTIR analysis was performed using a Spectrum Two FT-IR Spectrometer (PerkinElmer, Waltham, MA, USA) in the Attenuated Total Reflectance (ATR) mode. The spectrometer features were as follows: a high-performance, room temperature LiTaO3 MIR detector, a standard optical system with KBr windows for the data collection over a spectral range of 4000–350 cm−1 at a resolution of 0.5 cm−1. All the spectra were initially collected in the ATR mode and converted into the IR transmittance mode. FTIR measurements were conducted to ascertain the potential biomolecules and bonds that contribute to the structural and functional stabilization of Fmoc-FF hydrogel (indicated by the red line). The absorption bands of hydrogel in the IR spectrum were found within the amide band region; amide I is characterized by C=O stretching/hydrogen bonding with NH, amide II by N-H bending vibrations and C-N stretching vibrations, and amide III is associated with in-plane vibrations of C-N and N-H of the linked amide [Das 2017, Hanani 2011]. Figure SI displays the peaks for hydrogel at 3350 cm-1, which are attributed to the hydrogen bonding of water and amide-A. A peak around 1650 cm-1 corresponds to the emergence of amide-I in the gel. Fmoc-FF exhibits a peak at 1538 cm-1, indicative of amide-II. Peaks ranging from 1460 cm-1 to 1390 cm-1 in hydrogel can be ascribed to the symmetric and asymmetric bending vibrations of the methyl group. The C–O and C–O–C stretching, along with some C–OH stretching bands, were observed at 1015 cm-1, and 948 cm-1 [Lohrasbi 2020].

- Das, M. P., Suguna, P. R., Prasad, K. A. R. P. U. R. A. M., Vijaylakshmi, J. V., & Renuka, M. (2017). Extraction and characterization of gelatin: a functional biopolymer. Int. J. Pharm. Pharm. Sci, 9(9), 239.
- Nur Hanani ZA, Roos YH, Kerry JP. Fourier transform infrared (FTIR) spectroscopic analysis of biodegradable gelatin films immersed in water. International Congress on Engineering and Food, Proceedings; 2011
- Lohrasbi, S., Mirzaei, E., Karimizade, A., Takallu, S., & Rezaei, A. (2020). Collagen/cellulose nanofiber hydrogel scaffold: physical, mechanical and cell biocompatibility properties. Cellulose, 27, 927-940.

Table S1. The parameters of SICM and AFM experiments.

	**Young's modulus *E* determination using intrinsic force (colloidal pressure) by SICM**				Young's modulus *E* determination by AFM		
	**Capillary radius, R_{cap}, nm**	**Ion current, pA**	**Applied force, nN**	**Colloidal pressure, Pa**	Indenter radius, R_{tip}, nm	Applied force, nN	Pressure, Pa
MCF-7 on Petri dish	40-55	1800-2400	0,145	9920	70	0.19	1730
MCF-7 on Fmoc-FF	40-55	1800-2400	0,218	8990	70	0.186	1690
MCF-7 on Fmoc-FF+chitosan	40-55	1800-2400	0,22	9530	70	0.149	1355

Herein the colloidal pressure for SICM experiment was calculated using formula (S5) (see below) with the MCF-7 on Petri dish, MCF-7 on Fmoc-FF and MCF-7 on Fmoc-FF+chitosan deformations: 87, 130 and 170 nm, capillary radii were Rcap=48, 44 and 42 nm, correspondingly,. The applied pressures for AFM measurements were calculated for the 500 nm deformations for MCF-7 on Petri dish, MCF-7 on Fmoc-FF and MCF-7 on Fmoc-FF+chitosan samples.

The calculation of colloidal pressure in the SICM experiments

As it was previously mentioned the intrinsic force *F* between pipette and the object was initially obtained on the example of decan drop [Kolmogorov, et.al. 2021, Clarke, et.al. 2016]. *F* was performed from the assumption of surface tension force balance F_σ:

$$\vec{F} = 2\vec{F}_\sigma \quad \text{(S1)}$$

The contact radius between capillary and object according to the Hertz model for sphere can be calculated as follows:

$$a = \sqrt{R \cdot d} \quad \text{(S2)}$$

where *R* is inner radius of capillary and *d* is indentation of the object. The force of surface tension was calculated using the Laplace formula:

$$F_\sigma = 2\pi a\sigma \quad \text{(S3)}$$

The additional coefficient 2 was used as a radius of the pipette assuming that the whole radius is 2 times bigger than the inner radius of the pipette. So, the force applied by the pipette (capillary) looks as follows:

$$F = 4\pi\sigma\sqrt{R \cdot d} \quad \text{(S4)}$$

where σ is a surface tension parameter. In this experiment the value $\sigma = 0{,}25 \cdot 10^{-3}$ N/m [Adewunmi, et.al. 2019].

The colloidal pressure can be estimated as:

$$p = \frac{F}{S} = \frac{F}{\pi a^2} = \frac{F}{\pi R d} \quad \text{(S5)}$$

On the example of decan drop the dependence of surface deformation on tip radius was obtained. After that using formula (S4) the dependence of intrinsic force on tip radius was achieved.

In this theory it is assumed that the calibration graph, namely, the dependence of the intrinsic force on the pipette radius, can be used for different samples (cells, soft biomaterials), there is no direct contact between samples and the pipette and the interactions occurs through their double layers.

So, in the experiments with cells on Petri dish and cells on hydrogels the deformation of samples d was measured using pipette (capillaries) of certain radius R, after that the colloidal pressure p was calculated using formula (S5), where intrinsic force F was taken from calibration curve.

V. S. Kolmogorov, A. S. Erofeev, E. Woodcock, Y. M. Efremov, A. P. Iakovlev, N. A. Savin, A. V. Alova, S. V. Lavrushkina, I. I. Kireev, A. O. Prelovskaya, E. V. Sviderskaya, D. Scaini, N. L. Klyachko, P. S. Timashev, Y. Takahashi, S. V. Salikhov, Y. N. Parkhomenko, A. G. Majouga, C. R. W. Edwards, P. Novak, Y. E. Korchev, P. V. Gorelkin, *Nanoscale*, 2021, **13**, 6558.

R. W. Clarke, P. Novak, A. Zhukov, E. J. Tyler, M. Cano-Jaimez, A. Drews, D. Klenerman, *Soft Matter*, 2016, **12**, 7953.

- A. A. Adewunmi, M. S. Kamal, *Energy & Fuels*, 2019, *33*(9), 8456-8462

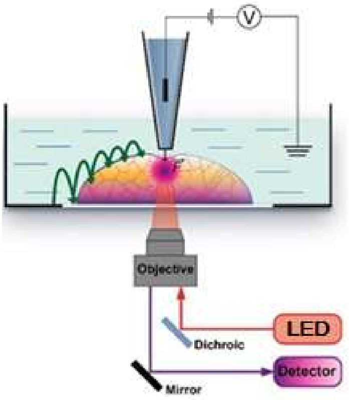

Figure S8. Schematic representation of SICM experimental setup that allows the simultaneous topographical, quantitative nanomechanical mapping and fluorescence imaging representation.

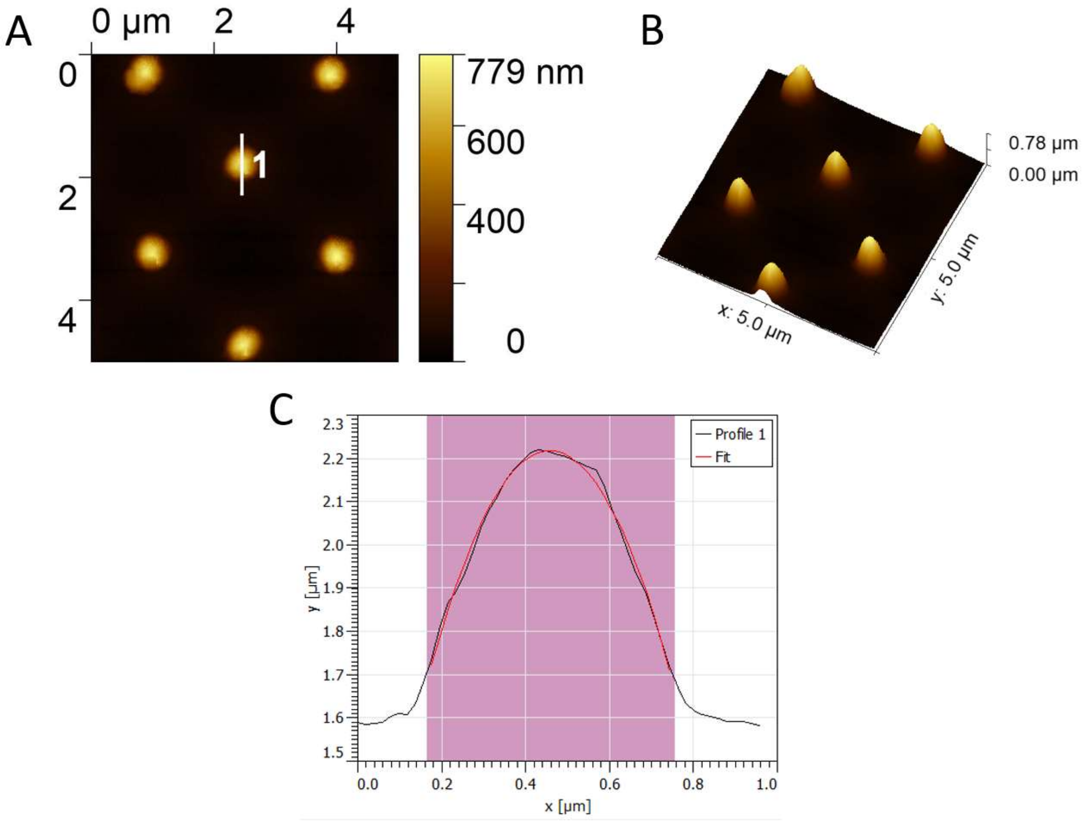

Figure S9. Estimation of the tip shape by scanning the TGT1 calibration grating. (A) Topography of TGT1 calibration grating acquired with the PFQNM-LC-A-CAL probe. (B) 3D representation of the same image. (C) Cross-section over one of the peaks fitted with a quadratic function (red curve).
